# Synthesis, crystal structure and Hirshfeld surface analysis of 5-cyclo­propyl-*N*-(2-hy­droxy­eth­yl)-1-(4-methyl­phen­yl)-1*H*-1,2,3-triazole-4-carboxamide

**DOI:** 10.1107/S2056989021009774

**Published:** 2021-09-28

**Authors:** Nazariy T. Pokhodylo, Yurii Slyvka, Volodymyr Pavlyuk

**Affiliations:** a Department of Organic Chemistry, Ivan Franko National University of Lviv, Kyryla i Mefodiya, 6, Lviv, 79005, Ukraine; b Department of Inorganic Chemistry, Ivan Franko National University of Lviv, Kyryla i Mefodiya, 6, Lviv, 79005, Ukraine

**Keywords:** crystal structure, 1,2,3-triazole, amide, 1,2,3-triazole-4-carboxamide, Hirshfeld surface analysis

## Abstract

The title compound, C_15_H_18_N_4_O_2_, was obtained *via* a two-step synthesis (Dimroth reaction and amidation) for anti­cancer activity screening and was selected from a 1*H*-1,2,3-triazole-4-carboxamide library. The cyclo­propyl ring is oriented almost perpendicular to the benzene ring [dihedral angle = 87.9 (1)°], while the dihedral angle between the mean plane of the cyclo­propyl ring and that of the triazole ring is 55.6 (1)°. In the crystal, the mol­ecules are linked by O—H⋯O and C—H⋯N inter­actions into infinite ribbons propagating in the [001] direction, which are inter­connected by weak C—H⋯O inter­actions into layers. The inter­molecular inter­actions were characterized *via* Hirshfeld surface analysis, which indicated that the largest fingerprint contact percentages are H⋯H (55.5%), N⋯H/H⋯N (15.4%), C⋯H/H⋯C (13.2%) and O⋯H/H⋯O (12.9%).

## Abstract 2

The title compound was obtained *via* a two-step synthesis (Dimroth reaction and amidation) for anti­cancer activity screening and was selected from a 1*H*-1,2,3-triazole-4-carboxamide library. The cyclo­propyl ring is oriented almost perpendicular to the benzene ring [dihedral angle = 87.9 (1)°], while the dihedral angle between the mean plane of the cyclo­propyl ring and that of the triazole ring is 55.6 (1)°. In the crystal, the mol­ecules are linked by O—H⋯O and C—H⋯N inter­actions into infinite ribbons propagating in the [001] direction, which are inter­connected by weak C—H⋯O inter­actions into layers.

## Chemical context   

The 1,2,3-triazolyl-4-carboxamide motif is of great inter­est in drug discovery, especially in relation to anti­cancer and anti­microbial research. Besides the well-known drugs rufinamide and carb­oxy­amido­triazole, several preclinical studies are ongoing. As an example of anti­tumour activity evaluations, libraries of 1,2,3-triazole-4-carboxamides containing podophyllotoxin (Reddy *et al.*, 2018 ▸), 1-*R*-*N*-[(1-*R*-1*H-*1,2,3-triazol-4-yl)meth­yl]-1*H-*1,2,3-triazole-4-carboxamides (Elamari *et al.*, 2013 ▸), 5-(tri­fluoro­meth­yl)-1*H*-1,2,3-triazole-4-carboxamides (Wang *et al.*, 2018 ▸; Zhou *et al.*, 2014 ▸) and 1-benz­yl-*N*-[2-(phenyl­amino)­pyridin-3-yl]-1*H-*1,2,3-triazole-4-carboxamides (Prasad *et al.*, 2019 ▸) have been tested. Several 1,4,5-tri­substituted 1,2,3-triazole-4-carboxamides showed high affinity in the nanomolar concentration range toward Hsp90 associated with cell proliferation inhibition (Taddei *et al.*, 2014 ▸; Giannini *et al.*, 2015 ▸). Moreover, 4-[4-(hydrazinecarbon­yl)-5-methyl-1*H*-1,2,3-triazol-1-yl]benzene­sulfonamide was found to act as a COX-2 inhibitor (Bekheit *et al.*, 2021 ▸).

In our previous studies, new active compounds with a 1,2,3-triazolyl-4-carboxamide motif were reported (Shyyka *et al.*, 2019 ▸; Pokhodylo, Shyyka, Finiuk & Stoika, 2020 ▸; Pokhodylo, Slyvka & Pavlyuk, 2020 ▸). Additionally, 1,2,3-triazolyl-4-carboxamide derivatives were found to be inhibitors of the Wnt/*β*-catenin signalling pathway (Obianom *et al.*, 2019 ▸). In addition, compounds with this motif exhibited fungicidal (Wang *et al.*, 2014 ▸), anti­viral (Krajczyk *et al.*, 2014 ▸) and anti­microbial (Pokhodylo *et al.*, 2021 ▸; Jadhav *et al.*, 2017 ▸) activities. The most convenient synthetic path to diverse 1*H-*1,2,3-triazole-4-carboxamides is a two-step synthesis involving the Dimroth reaction of organic azides with *β*-ketoesters (Pokhodylo & Obushak, 2019 ▸) followed by amidation of the resulting 1*H-*1,2,3-triazole-4-carb­oxy­lic acids.

Given the practical inter­est of 1-aryl-1*H*-1,2,3-triazole-4-carboxamides in anti­cancer and anti­microbial research, in the present paper, we report the mol­ecular and crystal structure of the title compound C_15_H_18_N_4_O_2_, highlighting its mol­ecular conformation and analysing the inter­molecular inter­actions. The cyclo­propyl substituent was selected as it meets the criteria of lead-oriented synthesis, increasing the number of *sp* ^3^-carbon atoms, but at the same time is conformationally restricted and occupies minimal volume among other C3-alkyl substituents. Moreover, the 5-cyclo­propyl­triazole fragment could appear as a bis­ected or perpendicular conformer.
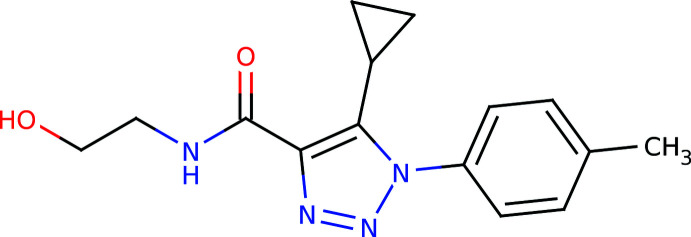


## Structural commentary   

**Figure 1:**
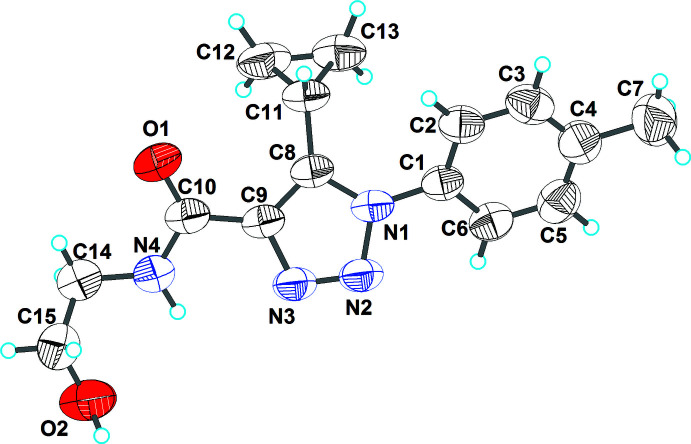
The mol­ecular structure of the title compound with displacement ellipsoids drawn at the 50% probability level.

The title compound crystallizes in the monoclinic centrosymmetric space group *P*2_1_/*c*, with one mol­ecule in the asymmetric unit as shown in Fig. 1 ▸. The mol­ecular structure possesses three conformational degrees of freedom due to free rotation about the C9—C10, C8—C11 and N1—C1 single bonds. The C10/N4/O1 amide group is turned slightly relative to the N1/N2/N3/C8/C9 triazole ring by 11.71 (4)°. Within the C11/C12/C13 cyclo­propyl ring, the C—C bond lengths differ by an insignificant amount [C11—C12 = 1.488 (3), C11—C13 = 1.492 (3), C12—C13 = 1.471 (3) Å]. The cyclo­propyl ring is oriented almost perpendicular to the C1–C6 benzene ring and the dihedral angle between these planes is 87.9 (1)°. The dihedral angle between the mean plane of the cyclo­propyl ring and that of the triazole ring is 55.6 (1)°.

A similar location of the cyclo­propyl ring relative to the 1,2,3-triazole ring was also observed in 5-cyclo­propyl-1-(3-meth­oxy­phen­yl)-1*H-*1,2,3-triazole-4-carb­oxy­lic acid (Pokhodylo *et al.*, 2017 ▸), but in the structure of the related compound *N*-(4-chloro­phen­yl)-5-cyclo­propyl-1-(4-meth­oxy­phen­yl)-1*H-*1,2,3-triazole-4-carboxamide (Pokhodylo & Slyvka *et al.*, 2020 ▸), the cyclo­propyl ring is close to coplanar with the aryl substituent. An intra­molecular N4—H4⋯N3 close contact (H⋯N = 2.37 Å; N—H⋯N = 106°) is observed.

The dihedral angle between the tolyl and 1,2,3-triazole rings in the title compound is 32.75 (7)°, which is comparable with the corresponding angle in 5-cyclo­propyl-1-(3-meth­oxy­phen­yl)-1*H-*1,2,3-triazole-4-carb­oxy­lic acid [39.1 (2)°] but lower than in the structure of 5-methyl-1-(4-nitro­phen­yl)-1*H*-1,2,3-triazol-4-yl­phospho­nate [45.36 (6)°] (Pokhodylo, Shykka, Goreshnik *et al.*, 2020 ▸). Conversely, in the triazoles unsubstituted at the 5-position, [1-(3-bromo- or 4-fluoro­phen­yl)-1*H*-1,2,3-triazol-4-yl]methyl methyl­phospho­nate, these angle are 22.9 (3) and 15.7 (2)°, respectively (Pokhodylo, Shyyka *et al.*, 2019 ▸).

## Supra­molecular features   

**Figure 2:**
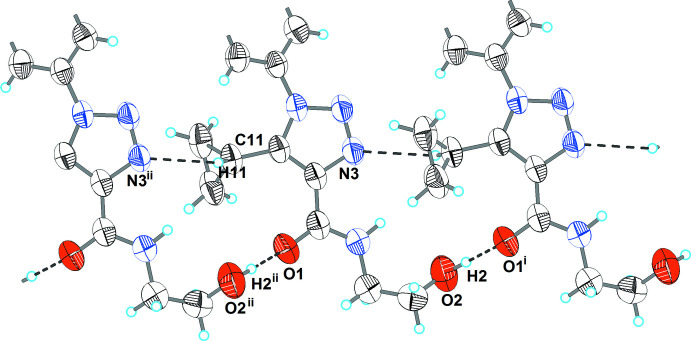
The hydrogen-bonded ribbon in the title compound. Hydrogen bonds are shown as dashed lines. The symmetry codes are as in Table 1 ▸.

**Figure 3:**
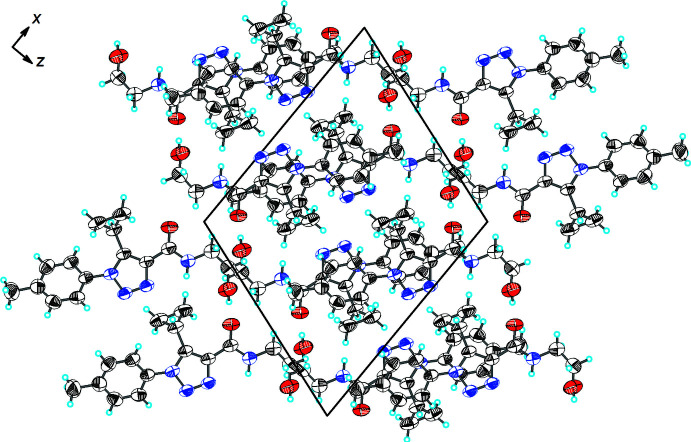
A view along the *b-*axis direction of the crystal packing of the title compound.

As shown in Fig. 2 ▸ and Table 1 ▸, the extended structure of the title compound features a number of directional inter­molecular inter­actions. The mol­ecules are linked by O2—H2⋯O1^i^ and C11—H11⋯N3^ii^ (see Table 1 ▸ for symmetry codes) inter­actions into an infinite ribbon propagating in the [001] direction. The ribbons are inter­connected by a weak C5—H5⋯O2^iii^ inter­action into layers (Fig. 3 ▸).

## Hirshfeld surface analysis   

**Figure 4:**
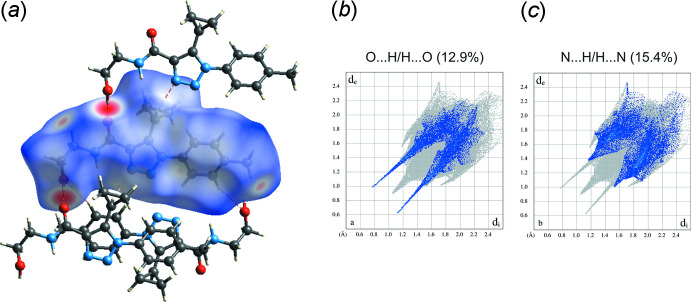
(*a*) Hirshfeld surface for the title compound mapped with *d*_norm_ over the range −0.68 to 1.46 showing the O—H⋯O, C—H⋯N and C—H⋯O hydrogen-bonded contacts. Fingerprint plots resolved into (*b*) O⋯H/H⋯O and (*c*) N⋯H/H⋯N contacts. Neighbouring mol­ecules associated with close contacts are also shown.

The significant inter­actions among the mol­ecules of the title compound can be visualized qualitatively through Hirshfeld surface analysis (Spackman & Jayatilaka, 2009 ▸). The mapping of the normalized contact distance (*d*_norm_) was performed using the *CrystalExplorer* software (Turner *et al.*, 2017 ▸). The most prominent inter­actions (short contact areas) are indicated on the Hirshfeld surfaces in red, whereas long contacts are shown in blue. Fingerprint plots were produced to show the inter­molecular surface bond distances with the regions highlighted for O⋯H/H⋯O and N⋯H/H⋯N inter­actions (Fig. 4 ▸). The contributions to the surface area for such contacts are 12.9% and 15.4%, respectively. The relatively low percentage of C⋯H/H⋯C contacts (13.2%) indicates the small contribution of C—H⋯π inter­actions for consolidating the crystal packing. The contribution to the surface area for H⋯H contacts is 55.5%.

## Database survey   

The most closest related compounds containing a similar 1-aryl-1*H-*1,2,3-triazole-4-carboxamide skeleton to the title compound but with different substituents on the amide are: *N*-(4-chloro­phen­yl)-5-cyclo­propyl-1-(4-meth­oxy­phen­yl)-1*H*-1,2,3-triazole-4-carboxamide (Pokhodylo & Slyvka *et al.*, 2020 ▸), (*S*)-1-(4-chloro­phen­yl)*-N-*(1-hy­droxy-3-phenyl­prop­an-2-yl)-5-methyl-1*H-*1,2,3-triazole-4-carboxamide (I) [Cambridge Structural Database (Version 2021.1; Groom *et al.*, 2016 ▸) refcode ZIPSEY; Shen *et al.*, 2013 ▸], 1-(4-chloro­phen­yl)-5-meth­yl*-N*-[(3-phenyl-1,2-oxazol-5-yl)meth­yl]-1*H-*1,2,3-triazole-4-carb­oxamide (II) (LELHOB; Niu *et al.*, 2013 ▸), (5-methyl-1-[8-(tri­fluoro­meth­yl)quinolin-4-yl]-1*H-*1,2,3-tri­az­ol-4-yl)morph­o­lino)­methanone (III) (LOHWIP; Anuradha *et al.*, 2008 ▸) and 1-(3-amino-5-(3-hy­droxy-3-meth­yl­but-1-yn-1-yl)phen­yl)*-N-*butyl-1*H-*1,2,3-triazole-4-carb­ox­amide (IV) (BEBJEZ; Li *et al.*, 2012 ▸).

Compounds (I) and (II) crystallize in the monoclinic crystal system with space groups *P*2_1_ and *P*2_1_/c, respectively, while compounds (III) and (IV) crystallize in the triclinic space group *P* 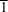. Structure (I) contains two crystallographically independent mol­ecules, the hydroxyl groups of which part­icipate in inter­molecular O—H⋯O hydrogen bonds. In contrast to the mol­ecular structure of title compound, the torsion angles between the phenyl rings and triazole rings in (I) are −45.2 (6)° (C5—C6—N1—N2) and 39.9 (6)° (C1′—C6′—N1′—N2′); the analogous value in (II) is 19.2 (2)°. In structure (II), the carboxamide groups connect neighbouring mol­ecules into infinite chains by means of N—H⋯O hydrogen bonds. The mol­ecules in structures (III) and (IV) are connected by N—H⋯O(oxazol) contacts. Similarly to (I) and (II), structure (III) contains a 5-methyl substituent at the triazole ring; as a result of the significant steric hindrance of 8-(tri­fluoro­meth­yl)quinoline, the dihedral angle between the rings is 54.7°. The phenyl and triazole rings in (IV) are close to coplanar (7.5°), while the hydroxyl, carboxamide and amino groups participate in O—H⋯O and N—H⋯O hydrogen bonds. Finally, two copper(I) π-complexes of compositions [Cu(C_12_H_13_N_5_O)(NO_3_)]·0.5H_2_O and [Cu(C_12_H_13_N_5_O)(CF_3_COO)](C_12_H_13_N_5_O is *N-*allyl-5-amino-1-phenyl-1*H-*1,2,3-tri­azole-4-carboxamide) were obtained by electrochemical synthesis (ZEQTOG and ZEQTUM; Slyvka *et al.*, 2012 ▸). Crystals of these compounds are monoclinic, space group *C*2/*c*: in both structures, the *N-*allyl-1*H-*1,2,3-triazole-4-carboxamide motif acts as a bridging chelating ligand and forms with the copper(I) atoms infinite chains containing [CuC_4_NO] seven-membered rings.

## Synthesis and crystallization   

**Figure 5:**
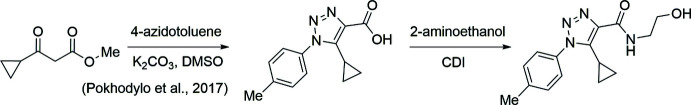
Synthesis of 5-cyclo­propyl-*N*-(2-hy­droxy­eth­yl)-1-(*p*-tol­yl)-1*H*-1,2,3-triazole-4-carboxamide.

5-Cyclo­propyl-1-*p*-tolyl-1*H*-1,2,3-triazole-4-carb­oxy­lic acid (Pokhodylo *et al.*, 2017 ▸) (1.22 g, 5.00 mmol) was added to a solution of 1,1′-carbonyl­diimidazole (CDI, 0.81 g, 5.0 mmol) in dry aceto­nitrile (5 ml) and the mixture was kept for 30 min at 323 K. Then, 0.3 ml of 2-amino­ethanol (0.31 g, 5.00 mmol) was added, and the mixture was heated at 343 K for 1 h. After cooling to room temperature, water (30 ml) was added. The precipitate was filtered off, washed with water on a filter, crystallized from diluted ethanol solution, and dried in air to give the title compound as colourless crystals, m.p. 396–397 K. The reaction scheme is shown in Fig. 5 ▸. IR (KBr, ν, cm^−1^): 1685 (C=O); 3370 (N—H). ^1^H NMR: (400 MHz, DMSO-*d*_6_): δ = 0.85–0.91 (*m*, 2H, CH_2_), 0.98–1.02 (*m*, 2H, CH_2_), 1.95–1.99 (*m*, 1H, CH), 2.46 (*c*, 3H, CH_3_), 3.37 (*q*, *J =* 5.8 Hz, 2H, CH_2_N), 3.54 (*q*, *J =* 5.8 Hz, 2H, CH_2_O), 4.58 (*t*, *J =* 6.0, Hz, 1H, OH), 7.37 (*d*, *J* = 7.6 Hz, 2H, H_Ar_-3,5), 7.43 (*d*, *J* = 7.6 Hz, 2H, H_Ar_-2,6), 8.14 (*t*, *J* = 5.4 Hz, 1H, NH). ^13^C NMR: (101 MHz, DMSO-*d*_6_): δ = 5.3 (CH), 8.2 (2 × CH_2_), 21.1 (CH_3_), 42.3 (CH_2_N), 59.5 (CH_2_O), 126.5 (2 × CH_Ar_-2,6), 130.1 (2 × CH_Ar_-3,5), 133.7 (C_Ar_-1), 137.2 (C_Triazole_-4), 139.2 (C_Ar_-4), 144.6 (C_Triazole_-5), 161.8 (C=O). MS, *m*/*z* = 287 (*M* ^+^+1). Calculated for C_15_H_18_N_4_O_2_, (%): C 62.92; H 6.34, N 19.57. Found (%): C 62.83; H 6.57, N 19.32.

## Refinement   

Crystal data, data collection and structure refinement details are summarized in Table 2 ▸. N-bound and O-bound H atoms were located in difference-Fourier maps and refined isotrop­ically. C-bound H atoms were positioned geometrically and refined using a riding model, with C—H = 0.93–0.98 Å and*U*_iso_(H) = 1.2*U*_eq_(C) or 1.5*U*_eq_(C-meth­yl).

## Supplementary Material

Crystal structure: contains datablock(s) I. DOI: 10.1107/S2056989021009774/hb7983sup1.cif

Structure factors: contains datablock(s) I. DOI: 10.1107/S2056989021009774/hb7983Isup2.hkl

Supporting information file. DOI: 10.1107/S2056989021009774/hb7983Isup3.mol

Supporting information file. DOI: 10.1107/S2056989021009774/hb7983Isup4.cml

CCDC reference: 736128

Additional supporting information:  crystallographic information; 3D view; checkCIF report

## Figures and Tables

**Table 1:** Hydrogen-bond geometry (Å, °)

*D*—H⋯*A*	*D*—H	H⋯*A*	*D*⋯*A*	*D*—H⋯*A*
O2—H2⋯O1^i^	0.95 (3)	1.78 (3)	2.734 (2)	177 (3)
C11—H11⋯N3^ii^	0.98	2.61	3.391 (2)	137
C5—H5⋯O2^iii^	0.93	2.66	3.564 (3)	164

Symmetry codes: (i) x, -y+{\script{1\over 2}}, z-{\script{1\over 2}}; (ii) x, -y+{\script{1\over 2}}, z+{\script{1\over 2}}; (iii) -x+1, -y+1, -z+1.

**Table 2:** Experimental details

Crystal data	
Chemical formula	C_15_H_18_N_4_O_2_
*M*_r_	286.33
Crystal system, space group	Monoclinic, *P*2_1_/*c*
Temperature (K)	293
*a*, *b*, *c* (Å)	14.3158 (6), 8.3972 (3), 13.0871 (4)
β (°)	108.040 (4)
*V* (Å^3^)	1495.90 (10)
*Z*	4
Radiation type	Mo *K*α
μ (mm^−1^)	0.09
Crystal size (mm)	0.5 × 0.4 × 0.06

Data collection	
Diffractometer	Oxford Diffraction Xcalibur3 CCD
Absorption correction	Multi-scan (*CrysAlis RED*; Oxford Diffraction, 2004 ▸)
*T*_min_, *T*_max_	0.935, 0.988
No. of measured, independent and observed [*I* > 2σ(*I*)] reflections	8400, 2632, 1621
*R*_int_	0.032
(sin θ/λ)_max_ (Å^−1^)	0.595

Refinement	
*R*[*F*^2^ > 2σ(*F* ^2^)], *wR*(*F* ^2^), *S*	0.047, 0.099, 1.05
No. of reflections	2632
No. of parameters	199
H-atom treatment	H atoms treated by a mixture of independent and constrained refinement
Δρ_max_, Δρ_min_ (e Å^−3^)	0.14, −0.20

Computer programs: *CrysAlis CCD* and *CrysAlis RED* (Oxford Diffraction, 2004 ▸), *SHELXT* (Sheldrick, 2015*a* ▸), *SHELXL* (Sheldrick, 2015*b* ▸) and *OLEX2* (Dolomanov *et al.*, 2009 ▸).

## supplementary crystallographic information

### Crystal data

**Table:**

C_15_H_18_N_4_O_2_	*F*(000) = 608
*M**_r_* = 286.33	*D*_x_ = 1.271 Mg m^−^^3^
Monoclinic, *P*2_1_/*c*	Mo *K*α radiation, λ = 0.71073 Å
*a* = 14.3158 (6) Å	Cell parameters from 1679 reflections
*b* = 8.3972 (3) Å	θ = 2.9–26.4°
*c* = 13.0871 (4) Å	µ = 0.09 mm^−^^1^
β = 108.040 (4)°	*T* = 293 K
*V* = 1495.90 (10) Å^3^	Lamina, clear colourless
*Z* = 4	0.5 × 0.4 × 0.06 mm

### Data collection

**Table:**

Oxford Diffraction Xcalibur3 CCD diffractometer	1621 reflections with *I* > 2σ(*I*)
ω scans	*R*_int_ = 0.032
Absorption correction: multi-scan (CrysAlis RED; Oxford Diffraction, 2004)	θ_max_ = 25.0°, θ_min_ = 2.9°
*T*_min_ = 0.935, *T*_max_ = 0.988	*h* = −17→17
8400 measured reflections	*k* = −9→9
2632 independent reflections	*l* = −15→8

### Refinement

**Table:**

Refinement on *F*^2^	Primary atom site location: dual
Least-squares matrix: full	Hydrogen site location: mixed
*R*[*F*^2^ > 2σ(*F*^2^)] = 0.047	H atoms treated by a mixture of independent and constrained refinement
*wR*(*F*^2^) = 0.099	*w* = 1/[σ^2^(*F*_o_^2^) + (0.035*P*)^2^ + 0.180*P*] where *P* = (*F*_o_^2^ + 2*F*_c_^2^)/3
*S* = 1.05	(Δ/σ)_max_ < 0.001
2632 reflections	Δρ_max_ = 0.14 e Å^−^^3^
199 parameters	Δρ_min_ = −0.20 e Å^−^^3^
0 restraints	

### Special details

**Table:**

Geometry. All esds (except the esd in the dihedral angle between two l.s. planes) are estimated using the full covariance matrix. The cell esds are taken into account individually in the estimation of esds in distances, angles and torsion angles; correlations between esds in cell parameters are only used when they are defined by crystal symmetry. An approximate (isotropic) treatment of cell esds is used for estimating esds involving l.s. planes.
Refinement. 1. Fixed Uiso At 1.2 times of: All C(H) groups, All C(H,H) groups At 1.5 times of: All C(H,H,H) groups 2.a Ternary CH refined with riding coordinates: C11(H11) 2.b Secondary CH2 refined with riding coordinates: C12(H12A,H12B), C13(H13A,H13B), C14(H14A,H14B), C15(H15A,H15B) 2.c Aromatic/amide H refined with riding coordinates: C2(H2A), C3(H3), C5(H5), C6(H6) 2.d Idealised Me refined as rotating group: C7(H7A,H7B,H7C)

### Fractional atomic coordinates and isotropic or equivalent isotropic displacement parameters (Å^2^)

**Table:**

	*x*	*y*	*z*	*U*_iso_*/*U*_eq_	
O1	0.13456 (11)	0.3294 (2)	0.60427 (12)	0.0974 (5)	
O2	0.06831 (14)	0.3928 (2)	0.21808 (14)	0.1042 (6)	
H2	0.092 (2)	0.318 (4)	0.177 (3)	0.171 (14)*	
N1	0.44653 (12)	0.35154 (18)	0.67420 (11)	0.0570 (4)	
N2	0.43955 (13)	0.3492 (2)	0.56776 (12)	0.0687 (5)	
N3	0.34622 (13)	0.3512 (2)	0.51377 (12)	0.0680 (5)	
N4	0.14491 (15)	0.3789 (3)	0.44072 (15)	0.0846 (6)	
H4	0.1840 (16)	0.397 (3)	0.4005 (17)	0.090 (7)*	
C1	0.54283 (14)	0.3518 (2)	0.75104 (14)	0.0573 (5)	
C2	0.56125 (17)	0.2755 (2)	0.84840 (16)	0.0660 (6)	
H2A	0.510965	0.223701	0.866145	0.079*	
C3	0.65554 (19)	0.2771 (3)	0.91933 (17)	0.0755 (6)	
H3	0.667851	0.226965	0.985570	0.091*	
C4	0.73224 (17)	0.3510 (3)	0.89461 (18)	0.0754 (6)	
C5	0.71121 (17)	0.4244 (3)	0.79535 (19)	0.0768 (6)	
H5	0.761557	0.474207	0.776551	0.092*	
C6	0.61784 (16)	0.4254 (2)	0.72389 (17)	0.0658 (5)	
H6	0.605361	0.475465	0.657611	0.079*	
C7	0.83533 (18)	0.3513 (4)	0.9729 (2)	0.1156 (10)	
H7A	0.853395	0.457928	0.997559	0.173*	
H7B	0.880342	0.311663	0.937725	0.173*	
H7C	0.837630	0.284323	1.033110	0.173*	
C8	0.35570 (14)	0.3551 (2)	0.68719 (14)	0.0546 (5)	
C9	0.29293 (14)	0.3532 (2)	0.58406 (14)	0.0583 (5)	
C10	0.18463 (16)	0.3519 (2)	0.54485 (16)	0.0671 (5)	
C11	0.33673 (14)	0.3634 (2)	0.79130 (14)	0.0616 (5)	
H11	0.329955	0.260032	0.823068	0.074*	
C12	0.27363 (19)	0.4924 (3)	0.81202 (17)	0.0882 (7)	
H12A	0.249069	0.572020	0.756496	0.106*	
H12B	0.228996	0.465129	0.851979	0.106*	
C13	0.37951 (19)	0.4924 (3)	0.87037 (17)	0.0791 (7)	
H13A	0.399859	0.464995	0.946092	0.095*	
H13B	0.419936	0.571906	0.850592	0.095*	
C14	0.03984 (18)	0.3804 (3)	0.38523 (18)	0.0979 (8)	
H14A	0.016031	0.489258	0.378087	0.117*	
H14B	0.005972	0.321759	0.427025	0.117*	
C15	0.01844 (17)	0.3080 (3)	0.27817 (18)	0.0946 (8)	
H15A	0.039484	0.197635	0.285111	0.114*	
H15B	−0.051722	0.310769	0.241585	0.114*	

### Atomic displacement parameters (Å^2^)

**Table:**

	*U*^11^	*U*^22^	*U*^33^	*U*^12^	*U*^13^	*U*^23^
O1	0.0884 (11)	0.1413 (16)	0.0775 (10)	−0.0021 (10)	0.0476 (9)	0.0143 (9)
O2	0.1267 (15)	0.1162 (15)	0.0851 (12)	−0.0073 (11)	0.0552 (11)	−0.0083 (10)
N1	0.0729 (11)	0.0568 (10)	0.0487 (9)	−0.0034 (8)	0.0296 (8)	−0.0040 (8)
N2	0.0769 (13)	0.0840 (13)	0.0532 (10)	−0.0042 (10)	0.0317 (9)	−0.0090 (9)
N3	0.0760 (13)	0.0828 (13)	0.0525 (9)	−0.0047 (10)	0.0302 (9)	−0.0081 (8)
N4	0.0674 (13)	0.1313 (18)	0.0594 (12)	−0.0073 (11)	0.0258 (10)	0.0050 (11)
C1	0.0700 (14)	0.0492 (12)	0.0575 (12)	0.0002 (10)	0.0271 (11)	−0.0064 (10)
C2	0.0834 (17)	0.0572 (13)	0.0609 (13)	−0.0025 (11)	0.0273 (12)	−0.0031 (10)
C3	0.0962 (19)	0.0669 (15)	0.0621 (13)	0.0136 (13)	0.0228 (14)	−0.0030 (11)
C4	0.0750 (16)	0.0746 (15)	0.0752 (15)	0.0140 (13)	0.0209 (12)	−0.0165 (13)
C5	0.0738 (17)	0.0738 (16)	0.0895 (17)	0.0014 (12)	0.0349 (14)	−0.0096 (13)
C6	0.0758 (15)	0.0599 (13)	0.0684 (13)	0.0026 (11)	0.0322 (12)	0.0003 (10)
C7	0.0830 (19)	0.155 (3)	0.1007 (19)	0.0267 (17)	0.0160 (15)	−0.0231 (18)
C8	0.0729 (13)	0.0448 (11)	0.0535 (11)	−0.0021 (10)	0.0304 (10)	−0.0007 (9)
C9	0.0719 (14)	0.0575 (13)	0.0538 (12)	−0.0048 (10)	0.0315 (10)	−0.0014 (10)
C10	0.0796 (15)	0.0702 (14)	0.0591 (13)	−0.0042 (12)	0.0328 (12)	−0.0001 (11)
C11	0.0893 (15)	0.0516 (12)	0.0536 (11)	−0.0055 (11)	0.0364 (10)	−0.0003 (10)
C12	0.116 (2)	0.0920 (18)	0.0739 (15)	0.0236 (15)	0.0541 (15)	0.0002 (12)
C13	0.116 (2)	0.0663 (15)	0.0657 (13)	−0.0099 (13)	0.0447 (14)	−0.0104 (11)
C14	0.0787 (18)	0.147 (2)	0.0738 (15)	0.0057 (15)	0.0315 (13)	−0.0002 (15)
C15	0.0722 (16)	0.125 (2)	0.0884 (18)	−0.0097 (14)	0.0268 (14)	−0.0072 (15)

### Geometric parameters (Å, º)

**Table:**

O1—C10	1.224 (2)	C6—H6	0.9300
O2—H2	0.95 (3)	C7—H7A	0.9600
O2—C15	1.408 (3)	C7—H7B	0.9600
N1—N2	1.3655 (19)	C7—H7C	0.9600
N1—C1	1.433 (2)	C8—C9	1.370 (2)
N1—C8	1.363 (2)	C8—C11	1.471 (2)
N2—N3	1.304 (2)	C9—C10	1.475 (3)
N3—C9	1.365 (2)	C11—H11	0.9800
N4—H4	0.89 (2)	C11—C12	1.488 (3)
N4—C10	1.324 (3)	C11—C13	1.492 (3)
N4—C14	1.454 (3)	C12—H12A	0.9700
C1—C2	1.377 (3)	C12—H12B	0.9700
C1—C6	1.378 (3)	C12—C13	1.471 (3)
C2—H2A	0.9300	C13—H13A	0.9700
C2—C3	1.381 (3)	C13—H13B	0.9700
C3—H3	0.9300	C14—H14A	0.9700
C3—C4	1.384 (3)	C14—H14B	0.9700
C4—C5	1.384 (3)	C14—C15	1.470 (3)
C4—C7	1.514 (3)	C15—H15A	0.9700
C5—H5	0.9300	C15—H15B	0.9700
C5—C6	1.374 (3)		
			
C15—O2—H2	108 (2)	N3—C9—C8	109.33 (17)
N2—N1—C1	117.80 (15)	N3—C9—C10	120.82 (17)
C8—N1—N2	110.87 (15)	C8—C9—C10	129.85 (16)
C8—N1—C1	131.32 (15)	O1—C10—N4	122.1 (2)
N3—N2—N1	106.95 (14)	O1—C10—C9	122.64 (19)
N2—N3—C9	109.13 (15)	N4—C10—C9	115.29 (17)
C10—N4—H4	119.3 (14)	C8—C11—H11	115.0
C10—N4—C14	124.32 (19)	C8—C11—C12	119.99 (17)
C14—N4—H4	116.4 (14)	C8—C11—C13	121.51 (16)
C2—C1—N1	121.04 (18)	C12—C11—H11	115.0
C2—C1—C6	120.41 (19)	C12—C11—C13	59.16 (14)
C6—C1—N1	118.51 (17)	C13—C11—H11	115.0
C1—C2—H2A	120.5	C11—C12—H12A	117.7
C1—C2—C3	119.0 (2)	C11—C12—H12B	117.7
C3—C2—H2A	120.5	H12A—C12—H12B	114.8
C2—C3—H3	119.1	C13—C12—C11	60.56 (14)
C2—C3—C4	121.9 (2)	C13—C12—H12A	117.7
C4—C3—H3	119.1	C13—C12—H12B	117.7
C3—C4—C7	121.3 (2)	C11—C13—H13A	117.7
C5—C4—C3	117.5 (2)	C11—C13—H13B	117.7
C5—C4—C7	121.2 (2)	C12—C13—C11	60.28 (14)
C4—C5—H5	119.2	C12—C13—H13A	117.7
C6—C5—C4	121.7 (2)	C12—C13—H13B	117.7
C6—C5—H5	119.2	H13A—C13—H13B	114.9
C1—C6—H6	120.2	N4—C14—H14A	109.5
C5—C6—C1	119.6 (2)	N4—C14—H14B	109.5
C5—C6—H6	120.2	N4—C14—C15	110.52 (19)
C4—C7—H7A	109.5	H14A—C14—H14B	108.1
C4—C7—H7B	109.5	C15—C14—H14A	109.5
C4—C7—H7C	109.5	C15—C14—H14B	109.5
H7A—C7—H7B	109.5	O2—C15—C14	109.4 (2)
H7A—C7—H7C	109.5	O2—C15—H15A	109.8
H7B—C7—H7C	109.5	O2—C15—H15B	109.8
N1—C8—C9	103.70 (15)	C14—C15—H15A	109.8
N1—C8—C11	124.99 (17)	C14—C15—H15B	109.8
C9—C8—C11	131.29 (17)	H15A—C15—H15B	108.3
			
N1—N2—N3—C9	−0.6 (2)	C2—C3—C4—C5	0.0 (3)
N1—C1—C2—C3	179.09 (16)	C2—C3—C4—C7	−179.8 (2)
N1—C1—C6—C5	−178.65 (17)	C3—C4—C5—C6	0.5 (3)
N1—C8—C9—N3	−1.0 (2)	C4—C5—C6—C1	0.0 (3)
N1—C8—C9—C10	178.57 (19)	C6—C1—C2—C3	1.5 (3)
N1—C8—C11—C12	125.2 (2)	C7—C4—C5—C6	−179.7 (2)
N1—C8—C11—C13	55.1 (3)	C8—N1—N2—N3	0.0 (2)
N2—N1—C1—C2	−146.14 (17)	C8—N1—C1—C2	34.7 (3)
N2—N1—C1—C6	31.5 (2)	C8—N1—C1—C6	−147.61 (19)
N2—N1—C8—C9	0.61 (19)	C8—C9—C10—O1	−10.8 (3)
N2—N1—C8—C11	−177.98 (16)	C8—C9—C10—N4	168.3 (2)
N2—N3—C9—C8	1.0 (2)	C8—C11—C12—C13	−111.0 (2)
N2—N3—C9—C10	−178.57 (17)	C8—C11—C13—C12	108.5 (2)
N3—C9—C10—O1	168.7 (2)	C9—C8—C11—C12	−53.0 (3)
N3—C9—C10—N4	−12.2 (3)	C9—C8—C11—C13	−123.1 (2)
N4—C14—C15—O2	−58.8 (3)	C10—N4—C14—C15	−141.9 (2)
C1—N1—N2—N3	−179.30 (16)	C11—C8—C9—N3	177.49 (18)
C1—N1—C8—C9	179.77 (18)	C11—C8—C9—C10	−3.0 (3)
C1—N1—C8—C11	1.2 (3)	C14—N4—C10—O1	−1.9 (3)
C1—C2—C3—C4	−1.0 (3)	C14—N4—C10—C9	179.0 (2)
C2—C1—C6—C5	−1.0 (3)		

### Hydrogen-bond geometry (Å, º)

**Table:**

*D*—H···*A*	*D*—H	H···*A*	*D*···*A*	*D*—H···*A*
O2—H2···O1^i^	0.95 (3)	1.78 (3)	2.734 (2)	177 (3)
C11—H11···N3^ii^	0.98	2.61	3.391 (2)	137
C5—H5···O2^iii^	0.93	2.66	3.564 (3)	164

Symmetry codes: (i) *x*, −*y*+1/2, *z*−1/2; (ii) *x*, −*y*+1/2, *z*+1/2; (iii) −*x*+1, −*y*+1, −*z*+1.
